# CONSORT-epidural dexmedetomidine improves gastrointestinal motility after laparoscopic colonic resection compared with morphine: Retraction

**DOI:** 10.1097/MD.0000000000025238

**Published:** 2021-03-19

The article “CONSORT-epidural dexmedetomidine improves gastrointestinal motility after laparoscopic colonic resection compared with morphine”,^[1]^ which was published in Volume 97, Issue 25 of *Medicine* is being retracted due to lack of proper ethical approval and trial registration. The paper shares the same ethical approval number and trial registration as an article previously published in *Plos One*.^[2]^ After investigation, it was determined that authors did not receive proper ethics approval and did not register the trial for the study published in *Medicine*.
